# Alcohol‐induced Wnt signaling inhibition during bone fracture healing is normalized by intermittent parathyroid hormone treatment

**DOI:** 10.1002/ame2.12116

**Published:** 2020-05-07

**Authors:** Esha M. Kapania, Taylor J. Reif, Aaron Tsumura, Jonathan M. Eby, John J. Callaci

**Affiliations:** ^1^ Internal Medicine‐Pediatric Resident Rush University Medical Center Chicago IL USA; ^2^ Limb Lengthening and Complex Reconstruction Fellowship Hospital for Special Surgery New York NY USA; ^3^ Department of Orthopaedic Surgery and Rehabilitation Loyola University Medical Center Maywood IL USA; ^4^ Alcohol Research Program (ARP) Loyola University Chicago Stritch School of Medicine Maywood IL USA

**Keywords:** ethanol, fracture callus, parathyroid hormone, Wnt signaling pathway

## Abstract

Nearly half of orthopaedic trauma patients are intoxicated at the time of injury, and excess alcohol consumption increases the risk for fracture nonunion. Previous studies show alcohol disrupts fracture associated Wnt signaling required for normal bone fracture repair. Intermittent parathyroid hormone (PTH) promotes bone growth through canonical Wnt signaling, however, no studies have investigated the effect of PTH on alcohol‐inhibited bone fracture repair. Male C57BL/6 mice received two‐3 day alcohol binges separated by 4 days before receiving a mid‐shaft tibia fracture. Postoperatively, mice received PTH daily until euthanasia. Wnt/β‐catenin signaling was analyzed at 9 days post‐fracture. As previously observed, acute alcohol exposure resulted in a >2‐fold decrease in total and the active form of β‐catenin and a 2‐fold increase in inactive β‐catenin within the fracture callus. Intermittent PTH abrogated the effect of alcohol on β‐catenin within the fracture callus. Upstream of β‐catenin, alcohol‐treated animals had a 2‐fold decrease in total LRP6, the Wnt co‐receptor, which was restored with PTH treatment. Alcohol nor PTH had any significant effect on GSK‐3β. These data show that intermittent PTH following a tibia fracture restores normal expression of Wnt signaling proteins within the fracture callus of alcohol‐treated mice.

## 1 INTRODUCTION

Fracture nonunion occurs in 5%‐10% of bone fractures, resulting in additional surgeries, prolonged patient recovery time and substantial healthcare costs.^1^, ^2^, ^3^ Therefore, the primary goal following a fracture is reducing the risk of fracture nonunion. Several risk factors, such as high body mass index, smoking and alcohol abuse increase the risk of a fracture progressing to a nonunion.^1^, ^4^ Alcohol abuse is a risk factor of particular interest as clinical studies have found that nearly half of orthopaedic trauma patients have elevated blood alcohol at the time of injury.^5^, ^6^ Additionally, previous studies in rodent models have found that binge alcohol consumption results in delayed fracture healing and reduced biomechanical strength of the callus tissue.^7^, ^8^, ^9^ Thus, there is a need for adjunctive therapies to promote bone healing in patients deemed high risk for nonunion or those showing radiographic signs of delayed union.

Parathyroid hormone (PTH) administered intermittently promotes bone growth and increases bone mass.^10^ Teriparatide, recombinant human PTH [1‐34], is currently FDA approved for the treatment of osteoporosis.^11^ There is also clinical interest for the use of PTH as an adjunct to fracture healing. Indeed, several animal studies have demonstrated the benefits of PTH on fracture callus size and biomechanical properties.^12^, ^13^, ^14^ A randomized controlled trial demonstrated PTH administration lead to significantly reduced healing time and improved functional status in osteoporotic pelvic fractures.^15^ However, the effect of PTH on fracture healing in alcohol‐treated animals has yet to be described.

Canonical Wnt signaling is important for bone development, homeostasis and fracture repair.^16^ Activation of the Wnt receptor results in the activation and stabilization of downstream effector, β‐catenin.^16^ Activated β‐catenin translocates to the nucleus and promotes the transcription of genes involved in bone formation and regeneration.^16^ Canonical Wnt/β‐catenin signaling tightly regulates the differentiation of mesenchymal stem cells into bone (osteoblasts)‐ and cartilage (chondrocytes)‐producing cells that initiate repair and make up the fracture callus.^17^, ^18^, ^19^ Our laboratory has previously shown that alcohol exposure in rodents prior to a fracture promotes the phosphorylation of β‐catenin, targeting the protein for degradation and reduces total β‐catenin in the fracture callus.^20^ As a result, alcohol inhibits callus formation and decreases the biomechanical strength of the fracture callus.^20^ Interestingly, intermittent PTH has been shown to activate Wnt signaling and promote differentiation of mesenchymal stem cells into osteochondral lineages.^21^, ^22^

Based on this information, the goal of this study was to investigate whether intermittent PTH administration can be used as a targeted molecular therapy to counteract the deleterious effects of alcohol on fracture callus formation. We hypothesize that intermittent PTH administration following a fracture would counteract the effect of alcohol on fracture healing by restoring Wnt/β‐catenin signaling during fracture repair.

## 2 METHODS

All animal procedures were approved by the Institutional Animal Care and Use Committee of Loyola University Chicago (Maywood, IL, USA) and compiled with the US National Research Council's Guide for the Care and Use of Laboratory Animals, the US Public Health Service's Policy on Humane Care and Use of Laboratory Animals, and Guide for the Care and Use of Laboratory Animals.

### 2.1 Binge alcohol model

**Figure 1:**
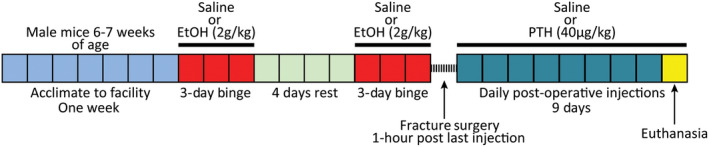
Binge alcohol paradigm and post‐operative treatment timeline. In a representative experiment, 5‐6 mice/group were administered saline or alcohol as shown before receiving a stabilized midshaft tibia fracture. Post‐operatively, animals received daily saline or parathyroid hormone until euthanasia

A total of 23 male C57BL/6 mice 6‐7 weeks of age were obtained from Jackson Laboratory (Bar Harbor, Maine) and acclimated to the facility for one week. The animal facility is a ventilated environment with a 12hour:12hour light‐dark cycle at a constant temperature of 21°C and the animals had unrestricted access to food and water. Animals were then randomly assigned to one of four treatment groups: Saline/Saline (n = 6), Alcohol/Saline (n = 5), Saline/PTH (n = 6), and Alcohol/PTH (n = 6). The nomenclature for each group corresponds to the pre‐fracture treatment (Saline or Alcohol) followed by the post‐fracture treatment (Saline or PTH). The treatment paradigm is summarized in Figure 1. Briefly, animals received 3 consecutive daily intraperitoneal injections of a 20% (vol/vol) ethanol/saline solution at a dose of 2 g/kg or equivalent volume of saline. Following 4 days of abstinence, the alcohol binge regimen was repeated to simulate a binge drinking pattern.^9^ At the time of fracture injury (1 hour after the last injection), blood alcohol average approximately 200 mg/dL.^23^

### 2.2 Fracture surgery protocol

**Figure 2:**
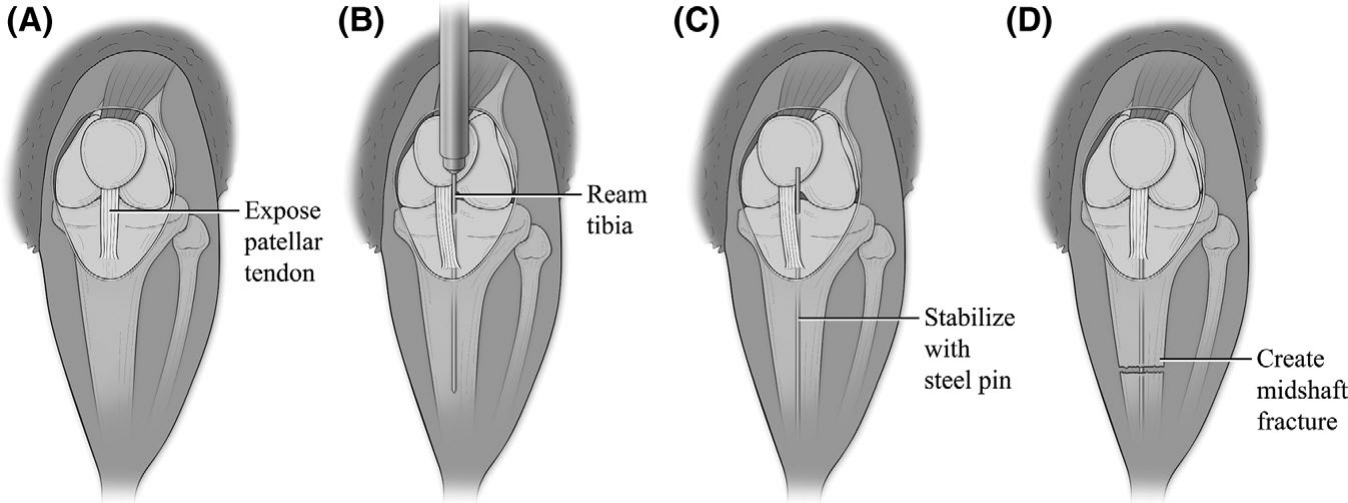
Anatomic illustrations depicting the stabilized mid‐shaft tibia fracture model. Figure modified from Bratton, et al^52^ A. Illustration showing the left hind stifle of C57BL/6 mouse. (A) small midline skin incision was made distal to the knee. Skin is manually adjusted to expose the knee joint and patellar tendon. (B) A 27‐gauge needle is slide behind the patellar tendon and inserted into the tibial plateau. Using a twisting motion, the needle reams through into the intramedullary canal. (C) The needle is removed and replaced with a steel insect pin that is inserted down the length of the tibia. (D) The insect pin is trimmed flushed with the tibia plateau. The skin incision is then manually adjusted to reveal the midshaft of the tibia. Using angled bone cutters, a complete fracture is made through both cortices of the diaphyseal bone while not damaging the intramedullary stabilizing pin. The incision is then suture closed after verifying the injury is complete and properly stabilized

One hour after receiving the final alcohol or saline injection, animals were administered an induction dose of anesthesia (0.5‐0.75 mg/kg ketamine and 0.06‐0.08 mg/kg xylazine, Patterson Veterinary, Greeley, CO) to facilitate hair removal from the left hind limb. Following hair removal, animals received subcutaneous prophylactic gentamycin (5 mg/kg, Patterson Veterinary, Greeley, CO) and were then placed on 1%‐2% vaporized isoflurane (Patterson Veterinary, Greeley, CO) for the duration of the fracture surgery. Under sterile conditions, a longitudinal incision was made over the left stifle and the patellar tendon exposed (Figure 2A). The tibia was reamed with a 27‐gauge needle (Figure 2B) prior to inserting a 0.25 mm stainless steel insect pin down the length of tibial canal (Figure 2C). A complete mid‐diaphyseal tibial fracture was created using angled bone scissors (Figure 2D) (Fine Science Tools, Foster City, CA). All animals received 1mg/kg long acting buprenorphine (Patterson Veterinary, Greeley, CO) by subcutaneous injection for post‐operative pain relief.

### 2.3 Post‐fracture PTH administration

All animals received daily intraperitoneal injections of human (1‐34) PTH (Bachem Inc, Bubendorf, Switzerland) or an equivalent volume of saline beginning at postoperative day (POD) one until euthanasia. The dose of 40 μg/kg PTH was selected based on published rodent PTH dosing regimens.^24^ Animals were humanely euthanized with CO_2_ and tibia specimens collected for molecular analysis on POD 9.

### 2.4 Molecular analysis

Tibia specimens were flash frozen in liquid nitrogen and stored at −80°C until use. Fracture callus tissue was separated from uninjured bone using a Dremel tool (Dremel Inc, Racine, WI, USA) and pulverized in lysis buffer (RIPA buffer, Halt phosphatase inhibitor cocktail [Sigma Aldrich, St. Louis, MO]) using a freezer mill (SPEX CertiPrep Inc, Metuchen, NJ). Total protein was measured using a Pierce BCA Protein Assay kit (Thermo Fisher Scientific Inc, Waltham, MA). Twenty micrograms of total protein from each sample was resolved on a 4%‐20% mini‐protean TGX Stain‐Free precast gel (Bio‐rad Inc, Hercules, CA). The total protein loaded was visualized using UV activation of the gel and analyzed with Image Lab software (Bio‐Rad, Inc, Hercules, CA), then transferred to PVDF membranes (Bio‐rad Inc, Hercules, CA). Membranes were probed with rabbit anti‐mouse total β‐catenin (Millipore 07‐1653, Billerica, MA), non‐phospho β‐catenin (Cell Signaling #19807, Danvers, MA), phospho‐β‐catenin (Cell Signaling #4176, Danvers, MA), total LRP6 (Cell Signaling #3395, Danvers, MA), total GSK‐3β (Abcam #131356, Cambridge, UK), phospho‐GSK‐3β (Ser9) (Cell Signaling #9336S, Danvers, MA) and phospho‐GSK‐3β (Y219) (Abcam #75745, Cambridge, UK). Visualization of bound antibodies was possible via secondary goat anti‐rabbit IgG (Abcam #ab6721, Cambridge, United Kingdom) and developed with SuperSignal West Pico chemiluminescent substrate (Thermo Fisher Scientific Inc, Waltham, MA, USA). Densitometric analysis was carried out utilizing the Image Lab software (Bio‐Rad, Inc, Hercules, CA, USA). Detected protein signals were normalized against the total protein concentration determined by Stain‐Free signal.

### 2.5 Statistical analysis

All data is expressed as the mean ± SEM and was analyzed using the SAS Version 9.4 (Cary, NC) statistical program. A Kruskal‐Wallis test was used to assess for overall variability in the ratio among the four experimental groups for each analysis set. A *P*‐value ≤.05 was noted as statistically significant.

## 3 RESULTS

### 3.1 Effects of binge alcohol and parathyroid hormone on the activation state of β‐catenin

**Figure 3:**
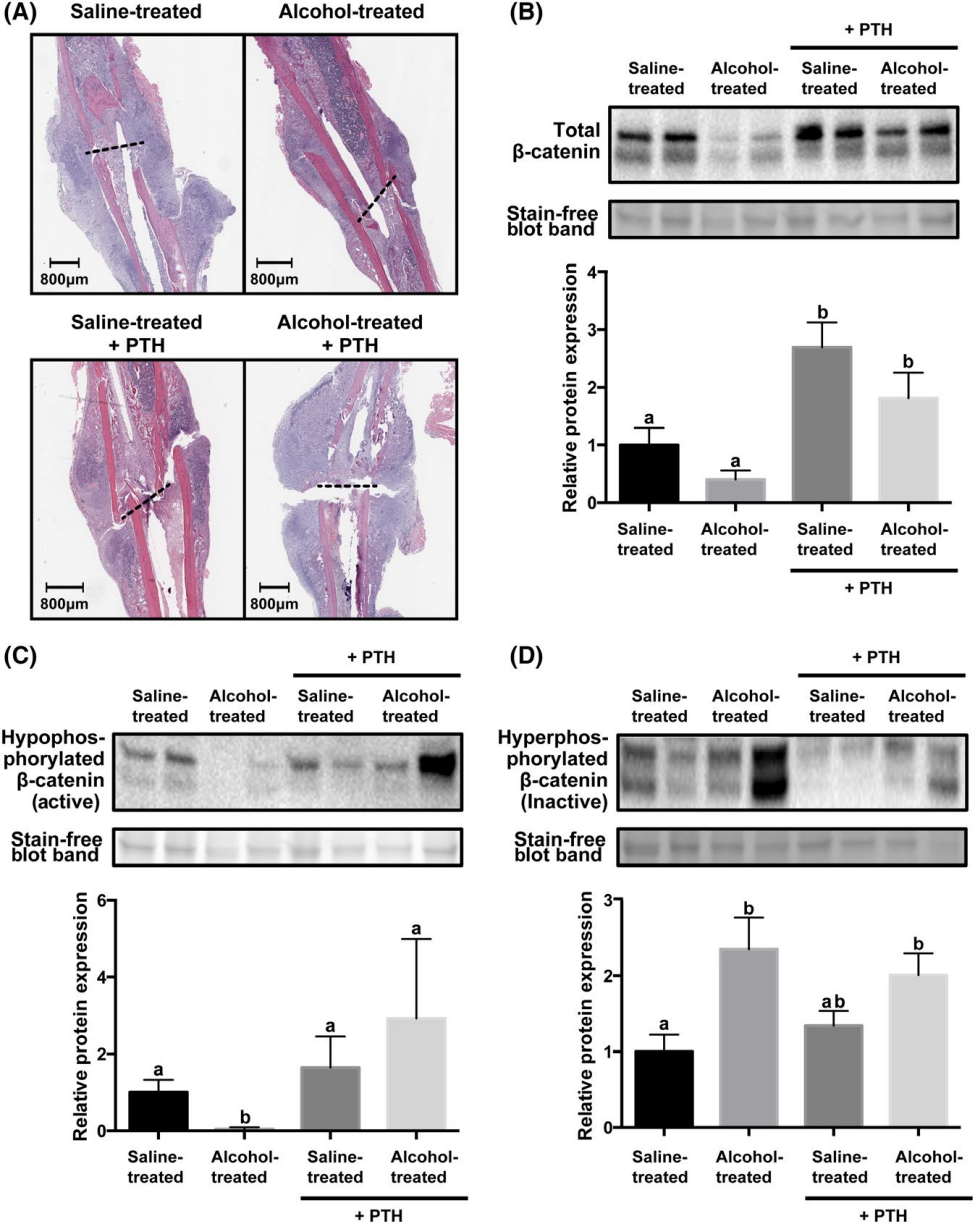
β‐catenin protein expression in callus tissue with or without intermittent parathyroid hormone treatment. (A) Representative hematoxylin and eosin stained histological samples from each treatment group. Fracture site is marked by a dashed black line. Western blot of (B) total β‐catenin; (C) hypophosphorylated (active) β‐catenin; and (D) hyperphosphorylated (inactive) β‐catenin. Corresponding quantification is graphed underneath each plot. Data are mean ± SEM are represented as the densitometric ratio of each experimental group relative to the saline‐treated animals. n = 5‐6, *P* < .05, Kruskal‐Wallis test. Groups not sharing a letter (eg a, b) are significant. Groups sharing a letter (eg a, a) are not significantly different

Canonical Wnt signaling is propagated through downstream effector protein β‐catenin. We have previously reported that alcohol‐treated animals have reduced fracture callus total β‐catenin compared to saline‐treated animals.^20^ In agreement with our previous studies, we observed callus total β‐catenin decreased 50% in alcohol‐treated animals as compared to saline‐treated animals (Figure 3A‐B). Parathyroid receptor activation has also been reported to stabilize β‐catenin and promote differentiation of mesenchymal stem cells into osteochondral lineages.^22^ Postoperative administration of PTH significantly increased callus total β‐catenin in both saline‐ and alcohol‐treated animals (Figure 3B, *P* = .017 and *P* = .016, respectively).

β‐catenin is regulated by phosphorylation. Hypophosphorylated β‐catenin is activated, promoting translocation of the protein to the nuclear to activate transcription, where hyperphosphorylated β‐catenin is inactive and targeted for degradation through the proteasome. In agreement with our previous findings, alcohol‐treated animals have a statistically significant reduction in callus hypophosphorylated β‐catenin (Figure 3C, *P* = .008) and a significant increase in hyperphosphorylated β‐catenin as compared to saline‐treated animals (Figure 3D, *P* = .03).^20^ Postoperative PTH treatment alone did not significantly increase hypophosphorylated β‐catenin when compared to saline‐treated animals (Figure 3C), however, levels of hypophosphorylated β‐catenin were restored to control levels in alcohol‐treated animals with postoperative PTH (Figure 3C, *P* = .010). In agreement with our past studies, alcohol‐treated animals had a significant increase in callus hyperphosphorylated β‐catenin as compared to saline‐treated animals (Figure 3D, *P* = .03).^20^ However, postoperative PTH did not significantly reduce hyperphosphorylated β‐catenin in the fracture callus of alcohol‐treated animals (Figure 3D).

### 3.2 Effects of binge alcohol and parathyroid hormone on the total LRP6

**Figure 4:**
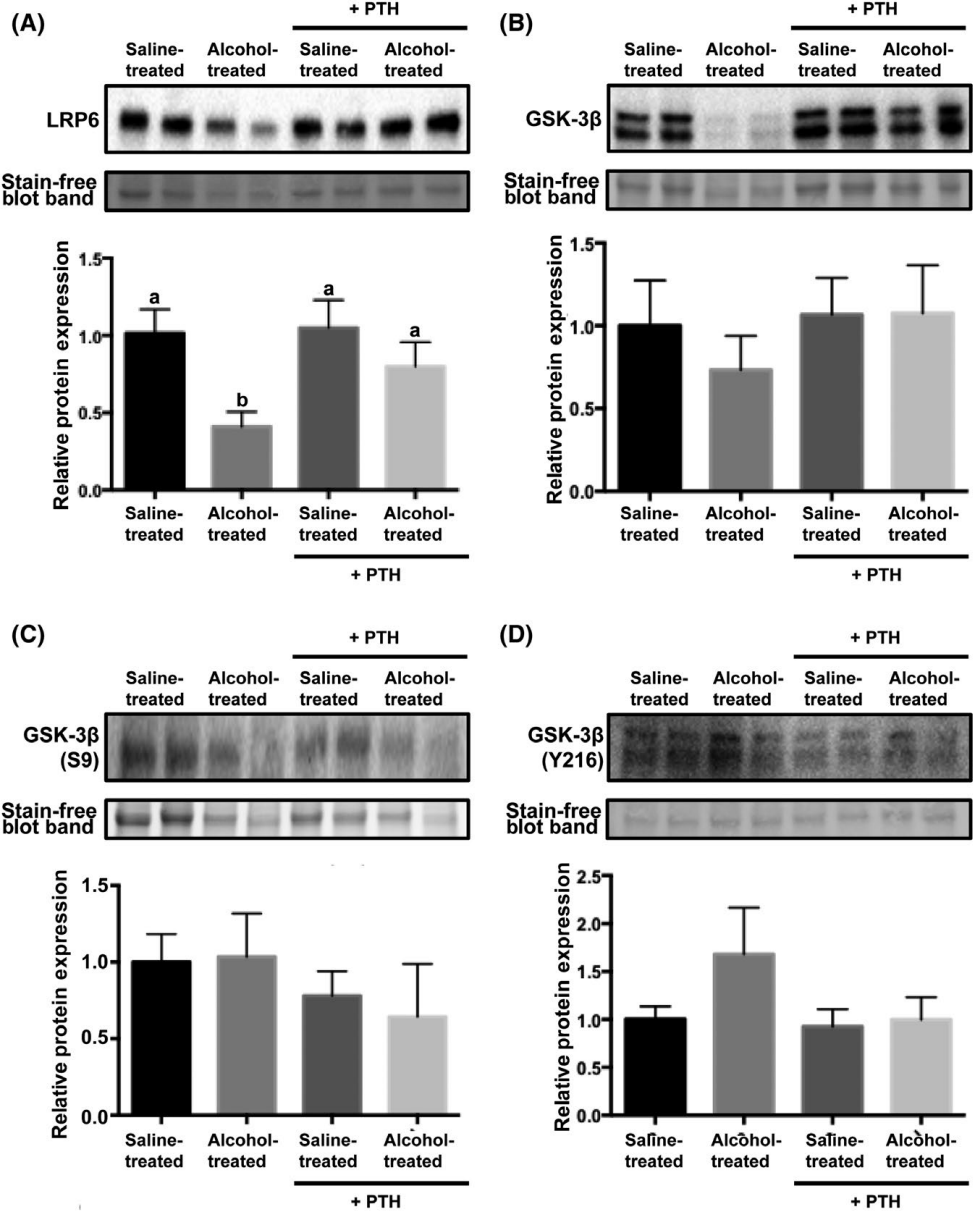
LRP6 and GSK‐3β protein expression in callus tissue with or without intermittent parathyroid hormone treatment. Western blot of (A) low‐density lipoprotein receptor‐related protein 6 (LRP6); (B) total Glycogen synthase kinase 3 beta (GSK‐3β); (C) inactive GSK‐3β (phospho‐S9); and (D) active GSK‐3β (phospho‐Y216). Corresponding quantification is graphed underneath each plot. Data are mean ± SEM are represented as the densitometric ratio of each experimental group relative to the saline‐treated animals. n = 5‐6, *P* < .05, Kruskal‐Wallis test. Groups not sharing a letter (eg a, b) are significant. Groups sharing a letter (eg a, a) are not significantly different

Low‐density lipoprotein receptor‐related protein 6 (LRP6) is a co‐receptor and key component in canonical Wnt receptor activation and signal propagation.^25^ We found that alcohol‐treated animals had a significant reduction in callus associated LRP6 as compared to saline‐treated animals (Figure 4A *P* = .01). Several studies have reported that LRP6 acts as a co‐receptor for the PTH receptor (PTHr).^22^, ^25^, ^26^ Binding of PTH to PTHr promotes the formation of PTHr/LRP6 complexes, which leads to rapid phosphorylation of LRP6 and activation of downstream β‐catenin signaling.^25^ To this end, we assessed whether PTH treatment restored LRP6 expression in the fracture callus of alcohol‐treated animals. We found that intermittent PTH administration rescued LRP6 expression in alcohol‐treated animals.

### 3.3 Effects of binge alcohol and parathyroid hormone on the activation state of GSK‐3β

Glycogen synthase kinase 3 beta (GSK‐3 β) through substrate‐mediated hyperphosphorylation of β‐catenin, is the principal negative regulator of canonical Wnt signaling.^27^ As shown in Figure 4B‐D, total GSK‐3β, inactive GSK‐3β (Phospho‐GSK‐3β (Ser9)) and active GSK‐3β (Phospho‐GSK‐3β (Y216)) were not significantly altered in the fracture callus of any treatment group.

## 4 DISCUSSION

Alcohol abuse has been implicated as a major risk factor for delayed fracture healing and nonunion in fracture repair.^4^, ^8^, ^28^, ^29^ This is of particular importance, as it is estimated that between 25%‐50% of orthopaedic trauma patients present acutely intoxicated.^5^, ^6^ Current options for treating fracture nonunion include internal and external fixation and autogenous or allogenic bone graft, however, each option has serious limitations. Thus, there is a significant need for adjunct therapeutics to surgical intervention which can support and promote fracture healing in individuals with an increased risk for nonunion. Intermittent PTH treatment has been reported to be well tolerated in animal and clinical studies, with supporting evidence for its role in fracture healing.^30^, ^31^, ^32^, ^33^, ^34^ The current investigation explored whether intermittent PTH could counteract the negative effects of alcohol on canonical Wnt/β‐catenin signaling in a binge alcohol model of deficient fracture repair. The Wnt/β‐catenin signaling pathway plays a pivotal role in fracture healing as it is required for the differentiation of mesenchymal stem cells into cartilage‐ and bone‐producing cells.^35^ Furthermore, it has been reported that β‐catenin signaling is tightly regulated in the early phases of fracture healing and alterations to β‐catenin signaling can have positive or negative effects on fracture healing.^35^ In this study, we found that intermittent PTH rescued several key components of callus associated canonical Wnt/β‐catenin signaling that were perturbed in alcohol‐treated animals.

Parathyroid hormone acts as an anabolic bone agent when delivered intermittently and several randomized controlled trials have been performed to determine its effect on fracture healing and spinal arthrodesis.^15^, ^36^, ^37^, ^38^, ^39^, ^40^ The results thus far have been equivocal. Peichl and Aspenberg *et al.* found PTH accelerated pelvic and distal radius fracture healing respectively^15^, ^36^ while Bhandari *et al.* and Johansson studying femoral neck and proximal humerus fractures did not find a benefit to union or improvement in patient reported outcomes, respectively.^38^, ^39^ Ebata *et al.* found weekly PTH injections significantly improved lumbar fusion rates in patients undergoing interbody fusion.^40^ Given the encouraging basic science and translational literature supporting PTH, the lack of significant side effects and the varied success in clinical investigations, interest in the drug as a tool to augment fracture healing remains strong.

Prior studies have shown that PTH activates Wnt signaling by increasing LRP6 receptor activation, β‐catenin activation and Wnt gene transcription,^21^ thereby activating differentiation of mesenchymal stem cells towards the osteochondral lineage.^22^, ^41^ Conversely, our laboratory has demonstrated that alcohol inhibits fracture callus formation and bone‐associated Wnt signaling, thus negatively affecting stem cell differentiation, osteoblast activity and bone formation.^20^, ^42^, ^43^, ^44^ Based on this information, we hypothesized that intermittent PTH treatment would normalize the effects of alcohol on Wnt signaling during fracture healing. We carried out our analysis on postoperative day 9 fracture callus tissue, which has been determined to be peak cartilaginous callus formation.^45^, ^46^ In agreement with our previous findings, we found that alcohol decreased fracture callus hypophosphorylated β‐catenin and increased hyperphosphorylated β‐catenin.^9^, ^20^, ^47^ These comparisons were used to experimental validate our present study. Alcohol‐treated animals receiving PTH had 65‐fold increase in hypophosphorylated, active β‐catenin as compared with animals treated with alcohol alone. These results are in agreement with prior studies that show PTH activates β‐catenin, and suggests that intermittent PTH stimulation can prevent the negative effects of alcohol on Wnt/β‐catenin signaling.^21^ When our alcohol‐treated mice were given PTH, we observed a recovery of total and hypophosphorylated β‐catenin expression in the fracture callus. These data suggests that the administration of PTH ameliorates the effect of alcohol on β‐catenin signaling in fracture callus of alcohol‐treated animals.

Administration of intermittent PTH has been found to stimulate bone formation.^24^, ^31^ Binding of PTH to the PTHr leads to PTHr and LRP6 complex formation on the cell surface.^22^, ^25^, ^26^ Previous studies have found that complex formation between PTHr and LRP6 leads to rapid activation of LRP6 and activation of downstream β‐catenin.^25^ Due to its role in stabilizing β‐catenin, LRP6 is of particular importance in the fracture repair pathway.^48^ Importantly, phosphorylated LRP6 has been found to be upregulated acutely during fracture healing.^35^ In our investigation we found a statistically significant decrease in the amount of LRP6 in alcohol‐treated animals as compared to the saline‐treated animals. To date, no prior studies have described the effect of alcohol on LRP6 expression within the fracture callus. We have previously reported that alcohol decreases LRP5 in the bones of alcohol‐treated mice.^49^ Combined with our current findings, our observations suggest that alcohol downregulates LRP5/6, which is elevated acutely in a fracture, inhibiting Wnt/ β‐catenin signaling. When alcohol‐treated animals are administered intermittent PTH, we observed a 2‐fold increase in callus LRP6 as compared to alcohol‐treated animals. These findings suggest that one mechanism by which intermittent PTH might rescue β‐catenin signaling within the fracture callus of alcohol‐treated animals is by upregulation of LRP6 expression. The observation that intermittent PTH does not further increase LRP6 expression in saline‐treated animals may suggest that LRP6 is already maximally expressed within the fracture callus during normal fracture healing.

β‐catenin is negatively regulated by the APC, axin and GSK‐3β complex by hyperphosphorylation, which targets β‐catenin to the proteasome for degradation.^50^ Within that complex, active GSK‐3β (phospho‐Y216) phosphorylates β‐catenin. Phosphorylated LRP6 has been shown to bind GSK‐3 complexes, inhibiting GSK‐3 phosphorylation of β‐catenin.^51^ We have previously reported that active GSK‐3β (phospho‐Y216) is increased in the fracture callus of alcohol‐treated animals, and that these effects were mitigated with post‐operative treatment with GSK‐3β inhibitor lithium chloride.^47^ We observed a similar trend in levels of active GSK‐3β (phospho‐Y216, p = 0.07)*[Correction added on 15 May 2020, after first online publication: The p‐value has been added]. in our alcohol‐treated animals as compared to saline‐treated animals. Further studies need to be done to determine if intermittent PTH reduces active GSK‐3β (phospho‐Y216) within the fracture callus of alcohol‐treated animals.

In a process that resembles endochondral ossification, mesenchymal stem cell‐derived chondrocytes and osteoblasts create a fracture callus following bone fracture. Previous studies show that intermittent PTH improves and accelerates fracture healing in part by increasing the size and improving the biomedical strength of the fracture callus,^12^, ^13^, ^14^, ^15^, ^30^, ^34^ suggesting intermittent PTH is directly stimulating chondrocyte and osteoblast activity. Indeed, several lines of evidence show that intermittent PTH enhances chondrogenesis and osteogenesis within the fracture callus.^22^, ^34^, ^41^ In our present study, we show that intermittent PTH rescues β‐catenin signaling in the fracture callus of alcohol treated rodents. Taken together, we hypothesize that impaired fracture callus healing in alcohol treated rodents can be rescued by intermittent PTH by enhancing chondrogenesis and osteogenesis with the fracture callus. Further studies are necessary to determine the effect of intermittent PTH on chondrogenesis and osteogenesis within the fracture callus of alcohol‐treated rodents.

The present study has its limitations. Most notably, this study was meant to serve as a preliminary study to determine whether intermittent PTH improved Wnt/β‐catenin signaling within the fracture callus of alcohol‐treated animals. Further research will be required to determine if the differences seen on the molecular level correlate to functional differences in the histology and biomechanics of the callus that forms during fracture healing. Additionally, this study was limited by the relatively small sample size of mice, leading to some of the variability in the dataset. Finally, the results of all the analyses are at a single time point in the complex process of fracture healing. Previous research show β‐catenin expression remains elevated for weeks after fracture, however, levels in a fracture callus reach maximal expression on post‐injury day 9.^35^ Having multiple post‐injury time points would have allowed for a temporal analysis into the effects of intermittent PTH on the fracture callus of alcohol‐treated animals.

In summary, the current work adds new information about the effects of intermittent PTH on Wnt/β‐catenin signaling within the fracture callus of alcohol‐treated animals. Intermittent PTH should continue to be investigated as a useful adjunct to fracture union given the scarcity of other clinically available systemic therapies.

## 5 CONCLUSIONS

Alcohol exposure inhibits Wnt signaling within the fracture callus. Administering intermittent PTH following a tibia fracture in alcohol‐treated mice restores normal expression of Wnt signaling proteins within the fracture callus.

## CONFLICT OF INTEREST

None.

## AUTHOR CONTRIBUTIONS

EMK and TJR performed and analyzed experiments. EMK, TJR, AT and JME analyzed data and participated in manuscript writing. All authors read and approved the final manuscript. All authors agree to be accountable for all aspects of the work.

## References

[1] Zura R , Xiong ZE , Einhorn T , et al. Epidemiology of fracture nonunion in 18 human bones. JAMA Surg. 2016;151:e162775. 27603155 10.1001/jamasurg.2016.2775

[2] Hak DJ , Fitzpatrick D , Bishop JA , et al. Delayed union and nonunions: epidemiology, clinical issues, and financial aspects. Injury. 2014;45:S3‐7. 10.1016/j.injury.2014.04.002 24857025

[3] Tay WH , de Steiger R , Richardson M , Gruen R , Balogh ZJ . Health outcomes of delayed union and nonunion of femoral and tibial shaft fractures. Injury. 2014;45:1653‐1658. 25062602 10.1016/j.injury.2014.06.025

[4] Kristensson H , Lunden A , Nilsson BE . Fracture incidence and diagnostic roentgen in alcoholics. Acta Orthop Scand. 1980;51:205‐207. 7435175 10.3109/17453678008990787

[5] Levy RS , Hebert CK , Munn BG , Barrack RL . Drug and alcohol use in orthopedic trauma patients: a prospective study. J Orthop Trauma. 1996;10:21‐27. 8926551 10.1097/00005131-199601000-00004

[6] Savola O , Niemela O , Hillbom M . Alcohol intake and the pattern of trauma in young adults and working aged people admitted after trauma. Alcohol Alcohol. 2005;40:269‐273. 15870091 10.1093/alcalc/agh159

[7] Elmali N , Ertem K , Ozen S , et al. Fracture healing and bone mass in rats fed on liquid diet containing ethanol. Alcohol Clin Exp Res. 2002;26:509‐513. 11981127

[8] Janicke‐Lorenz J , Lorenz R . Alcoholism and fracture healing. A radiological study in the rat. Arch Orthop Trauma Surg. 1984;103:286‐289. 6542345 10.1007/BF00387336

[9] Volkmer DL , Sears B , Lauing KL , et al. Antioxidant therapy attenuates deficient bone fracture repair associated with binge alcohol exposure. J Orthop Trauma. 2011;25:516‐521. 21738068 10.1097/BOT.0b013e31821f65cc PMC3139786

[10] Hock JM , Gera I . Effects of continuous and intermittent administration and inhibition of resorption on the anabolic response of bone to parathyroid hormone. J Bone Miner Res. 1992;7:65‐72. 1532281 10.1002/jbmr.5650070110

[11] Migliore A , Massafra U , Capuano A , Martin SM . Combined use of teriparatide and TNFalpha blockade: safety. Aging Clin Exp Res. 2007;19:18‐20. 18180602

[12] Andreassen TT , Fledelius C , Ejersted C , Oxlund H . Increases in callus formation and mechanical strength of healing fractures in old rats treated with parathyroid hormone. Acta Orthop Scand. 2001;72:304‐307. 11480610 10.1080/00016470152846673

[13] Andreassen TT , Ejersted C , Oxlund H . Intermittent parathyroid hormone (1–34) treatment increases callus formation and mechanical strength of healing rat fractures. J Bone Miner Res. 1999;14:960‐968. 10352105 10.1359/jbmr.1999.14.6.960

[14] Holzer G , Majeska RJ , Lundy MW , Hartke JR , Einhorn TA . Parathyroid hormone enhances fracture healing. A preliminary report. Clin Orthop Relat Res. 1999;366:258‐263. 10.1097/00003086-199909000-00033 10627743

[15] Peichl P , Holzer LA , Maier R , Holzer G . Parathyroid hormone 1–84 accelerates fracture‐healing in pubic bones of elderly osteoporotic women. J Bone Joint Surg Am. 2011;93:1583‐1587. 21915572 10.2106/JBJS.J.01379

[16] Baron R , Kneissel M . WNT signaling in bone homeostasis and disease: from human mutations to treatments. Nat Med. 2013;19:179‐192. 23389618 10.1038/nm.3074

[17] Hill TP , Spater D , Taketo MM , Birchmeier W , Hartmann C . Canonical Wnt/beta‐catenin signaling prevents osteoblasts from differentiating into chondrocytes. Dev Cell. 2005;8:727‐738. 15866163 10.1016/j.devcel.2005.02.013

[18] Day TF , Guo X , Garrett‐Beal L , Yang Y . Wnt/beta‐catenin signaling in mesenchymal progenitors controls osteoblast and chondrocyte differentiation during vertebrate skeletogenesis. Dev Cell. 2005;8:739‐750. 15866164 10.1016/j.devcel.2005.03.016

[19] Baksh D , Boland GM , Tuan RS . Cross‐talk between Wnt signaling pathways in human mesenchymal stem cells leads to functional antagonism during osteogenic differentiation. J Cell Biochem. 2007;101:1109‐1124. 17546602 10.1002/jcb.21097

[20] Lauing KL , Roper PM , Nauer RK , Callaci JJ . Acute alcohol exposure impairs fracture healing and deregulates beta‐catenin signaling in the fracture callus. Alcohol Clin Exp Res. 2012;36:2095‐2103. 22691115 10.1111/j.1530-0277.2012.01830.x PMC3443513

[21] Kulkarni NH , Halladay DL , Miles RR , et al. Effects of parathyroid hormone on Wnt signaling pathway in bone. J Cell Biochem. 2005;95:1178‐1190. 15962290 10.1002/jcb.20506

[22] Yu B , Zhao X , Yang C , et al. Parathyroid hormone induces differentiation of mesenchymal stromal/stem cells by enhancing bone morphogenetic protein signaling. J Bone Miner Res. 2012;27:2001‐2014. 22589223 10.1002/jbmr.1663 PMC3423493

[23] Penland S , Hoplight B , Obernier J , Crews FT . Effects of nicotine on ethanol dependence and brain damage. Alcohol. 2001;24:45‐54. 11524181 10.1016/s0741-8329(01)00142-2

[24] Chung DJ , Castro CHM , Watkins M , et al. Low peak bone mass and attenuated anabolic response to parathyroid hormone in mice with an osteoblast‐specific deletion of connexin43. J Cell Sci. 2006;119:4187‐4198. 16984976 10.1242/jcs.03162

[25] Wan M , Yang C , Li J , et al. Parathyroid hormone signaling through low‐density lipoprotein‐related protein 6. Genes Dev. 2008;22:2968‐2979. 18981475 10.1101/gad.1702708 PMC2577789

[26] Revollo L , Kading J , Jeong SY , et al. N‐cadherin restrains PTH activation of Lrp6/beta‐catenin signaling and osteoanabolic action. J Bone Miner Res. 2015;30:274‐285. 25088803 10.1002/jbmr.2323 PMC4315770

[27] Wu D , Pan W . GSK3: a multifaceted kinase in Wnt signaling. Trends Biochem Sci. 2010;35:161‐168. 19884009 10.1016/j.tibs.2009.10.002 PMC2834833

[28] Chakkalakal DA , Novak JR , Fritz ED , et al. Inhibition of bone repair in a rat model for chronic and excessive alcohol consumption. Alcohol. 2005;36:201‐214. 16377462 10.1016/j.alcohol.2005.08.001

[29] Nyquist F , Berglund M , Nilsson BE , Obrant KJ . Nature and healing of tibial shaft fractures in alcohol abusers. Alcohol Alcohol. 1997;32:91‐95. 9131898 10.1093/oxfordjournals.alcalc.a008240

[30] Kumabe Y , Lee SY , Waki T , et al. Triweekly administration of parathyroid hormone (1–34) accelerates bone healing in a rat refractory fracture model. BMC Musculoskelet Disord. 2017;18:545. 29268728 10.1186/s12891-017-1917-2 PMC5740882

[31] Datta NS . Osteoporotic fracture and parathyroid hormone. World J Orthop. 2011;2:67‐74. 22474638 10.5312/wjo.v2.i8.67 PMC3302045

[32] Cipriano CA , Issack PS , Shindle L , Werner CM , Helfet DL , Lane JM . Recent advances toward the clinical application of PTH (1–34) in fracture healing. HSS J. 2009;5:149‐153. 19290582 10.1007/s11420-009-9109-8 PMC2744747

[33] Tzioupis CC , Giannoudis PV . The safety and efficacy of parathyroid hormone (PTH) as a biological response modifier for the enhancement of bone regeneration. Curr Drug Saf. 2006;1:189‐203. 18690930 10.2174/157488606776930571

[34] Barnes GL , Kakar S , Vora S , Morgan EF , Gerstenfeld LC , Einhorn TA . Stimulation of fracture‐healing with systemic intermittent parathyroid hormone treatment. J Bone Joint Surg Am. 2008;90:120‐127. 18292366 10.2106/JBJS.G.01443

[35] Chen Y , Whetstone HC , Lin AC , et al. Beta‐catenin signaling plays a disparate role in different phases of fracture repair: implications for therapy to improve bone healing. PLoS Medicine. 2007;4:e249. 17676991 10.1371/journal.pmed.0040249 PMC1950214

[36] Aspenberg P , Genant HK , Johansson T , et al. Teriparatide for acceleration of fracture repair in humans: a prospective, randomized, double‐blind study of 102 postmenopausal women with distal radial fractures. J Bone Miner Res. 2010;25 404‐414. 19594305 10.1359/jbmr.090731

[37] Almirol EA , Chi LY , Khurana B , et al. Short‐term effects of teriparatide versus placebo on bone biomarkers, structure, and fracture healing in women with lower‐extremity stress fractures: a pilot study. J Clin Transl Endocrinol. 2016;5:7‐14. 29067229 10.1016/j.jcte.2016.05.004 PMC5644467

[38] Bhandari M , Jin L , See K , et al. Does Teriparatide improve femoral neck fracture healing: results from a randomized placebo‐controlled trial. Clin Orthop Relat Res. 2016;474:1234‐1244. 26932738 10.1007/s11999-015-4669-z PMC4814417

[39] Johansson TPTH . 1–34 (teriparatide) may not improve healing in proximal humerus fractures. A randomized, controlled study of 40 patients. Acta Orthop. 2016;87:79‐82. 26179771 10.3109/17453674.2015.1073050 PMC4940597

[40] Ebata S , Takahashi J , Hasegawa T , et al. Role of weekly teriparatide administration in osseous union enhancement within six months after posterior or transforaminal lumbar interbody fusion for osteoporosis‐associated lumbar degenerative disorders: a multicenter, prospective randomized study. J Bone Joint Surg Am. 2017;99:365‐372. 28244906 10.2106/JBJS.16.00230

[41] Tian Y , Xu Y , Fu Q , He M . Parathyroid hormone regulates osteoblast differentiation in a Wnt/beta‐catenin‐dependent manner. Mol Cell Biochem. 2011;355:211‐216. 21533763 10.1007/s11010-011-0856-8

[42] Chen Y , Chen L , Yin Q , et al. Reciprocal interferences of TNF‐alpha and Wnt1/beta‐catenin signaling axes shift bone marrow‐derived stem cells towards osteoblast lineage after ethanol exposure. Cell Physiol Biochem. 2013;32:755‐765. 24080828 10.1159/000354477

[43] Xu CQ , de la Monte SM , Tong M , Huang CK , Kim M . Chronic ethanol‐induced impairment of Wnt/beta‐catenin signaling is attenuated by PPAR‐delta agonist. Alcohol Clin Exp Res. 2015;39:969‐979. 25903395 10.1111/acer.12727 PMC4452420

[44] Wang Q , Song JW , Liu Y , Zhao XX . Involvement of Wnt pathway in ethanol‐induced inhibition of mouse embryonic stem cell differentiation. Alcohol. 2017;58:13‐18. 28109343 10.1016/j.alcohol.2016.11.006

[45] Hiltunen A , Vuorio E , Aro HT . A standardized experimental fracture in the mouse tibia. J Orthop Res. 1993;11:305‐312. 8483044 10.1002/jor.1100110219

[46] Le AX , Miclau T , Hu D , Helms JA . Molecular aspects of healing in stabilized and non‐stabilized fractures. J Orthop Res. 2001;19:78‐84. 11332624 10.1016/S0736-0266(00)00006-1

[47] Lauing KL , Sundaramurthy S , Nauer RK , Callaci JJ . Exogenous activation of Wnt/beta‐catenin signaling attenuates binge alcohol‐induced deficient bone fracture healing. Alcohol Alcohol. 2014;49:399‐408. 24627571 10.1093/alcalc/agu006 PMC4060733

[48] Zeng X , Huang H , Tamai K , et al. Initiation of Wnt signaling: control of Wnt coreceptor Lrp6 phosphorylation/activation via frizzled, dishevelled and axin functions. Development. 2008;135:367‐375. 18077588 10.1242/dev.013540 PMC5328672

[49] Himes R , Wezeman FH , Callaci JJ . Identification of novel bone‐specific molecular targets of binge alcohol and ibandronate by transcriptome analysis. Alcohol Clin Exp Res. 2008;32:1167‐1180. 18537941 10.1111/j.1530-0277.2008.00736.x PMC2728683

[50] Yost C , Torres M , Miller JR , Huang E , Kimelman D , Moon RT . The axis‐inducing activity, stability, and subcellular distribution of beta‐catenin is regulated in Xenopus embryos by glycogen synthase kinase 3. Genes Dev. 1996;10:1443‐1454. 8666229 10.1101/gad.10.12.1443

[51] Cselenyi CS , Jernigan KK , Tahinci E , Thorne CA , Lee LA , Lee E . LRP6 transduces a canonical Wnt signal independently of Axin degradation by inhibiting GSK3's phosphorylation of beta‐catenin. Proc Natl Acad Sci USA. 2008;105:8032‐8037. 18509060 10.1073/pnas.0803025105 PMC2430354

[52] Bratton A , Eisenberg J , Vuchkovska A , Roper P , Callaci JJ . Effects of Episodic Alcohol Exposure on BMP2 Signaling During Tibia Fracture Healing. J Orthop Trauma. 2018;32:288‐295. 29672339 10.1097/BOT.0000000000001160 PMC7485276

